# Policy analysis in sport: a review of mainstream meso-level frameworks and developing more sustainable policy for grassroots sport

**DOI:** 10.3389/fspor.2025.1529906

**Published:** 2025-02-06

**Authors:** Charles Mountifield

**Affiliations:** Faculty of Health, University of Canberra, Canberra, ACT, Australia

**Keywords:** implementation, bottom-up, policy frameworks, sport, top-down

## Abstract

This paper reviews some mainstream meso-level policy analysis frameworks widely applied in sport. There is, however, an absence of consensus for an established framework for analysing sport policy in general and, instead, techniques emanating from other fields of study have been relied upon. The resultant approach to sport policy analysis is inconsistent, multidimensional, and lacks unanimity, leading to calls for a sport-specific framework. This research outlines how meso-level frameworks have been applied in sport policy and issues linked to sustainability from a grassroots policy perspective. A narrative literature review provides an overview of prevalent approaches, namely Institutional Analysis, the Multiple Streams Framework, Policy Network Theory, and the Advocacy Coalition Framework. Aspects of applying these models to sport policy—including some key advantages and disadvantages—are outlined, especially the issue of conventional top-policy processes, the impact on policy implementers at grassroots level, and the potential for bottom-up policy influence. The article examines the four frameworks in the context of praxes in sport, noting the overall importance of a meso-level approach to sport policy analysis and that arriving at an holistic and inclusive accord has merit.

## Introduction

The sport sector is an increasingly complex, multifaceted and dynamic area (1–3) and is accompanied by a growing government influence in sport policy (4–6). The literature on sport policy, however, indicates a dearth of development in policy analysis frameworks that target sport exclusively. Indeed, while the application of frameworks is fundamental to research in sport (7–12), an absence of theoretical improvement of policy analysis frameworks in sport is evident (4, 13, 14). Similarly, there is only limited research in relation to the complexities of policy implementation—defined as “the process of interaction between the setting of goals and the actions geared to achieve them” (15)—in sport at grassroots level (16–19). For the purposes herein, grassroots sport is defined as the elementary layer of sport (20) taking place at the community level (21), and where there is evidence of the complexities associated with sport policy implementation (22–24).

The resultant effect of the sport policy situation is that topical analysis relies on frameworks drawn from other disciplines (4, 25, 26). Indeed, the trend has been toward adapting methods for sport policy analysis that are ordinarily applied in a non-sporting context (14). This scenario leads to issues with an inconsistent approach to policy analysis in sport, including the experiences at ground level (16, 27), partly because of the typically top-down nature of the policy process (28, 29). Scant attention has been given to the sport policy process, especially the reality of policy implementation (16, 27, 30, 31), including identifying causal mechanisms or independent variables that impact implementation performance (32, 33). With issues such as a reliance on policy frameworks from non-sporting disciplines, discrepancies with the policy process, and implementation concerns, there is merit in exploring a greater understanding of the impact of policy frameworks, especially from the perspective of the sustainability of grassroots sport.

## Significance

Policy implementation in sport, especially that at grassroots level, involves a series of issues for those involved in the policy process (16, 24, 34) and in some situations, leads to a paradox as a result of differing stakeholder priorities (20). Such a situation points to a degree of friction in relation to conventional top-down policy processes, the impact on policy implementers at grassroots level, and the potential for a more consistent and holistic application of policy (35–37). The significance of the situation is such that there have been calls to develop a unique framework for sport policy analysis (14). From a governance perspective, policy analysis is an essential tool for managing sport (12), and there is a need to structure policy in ways that enhance the potential for sport to benefit individuals and communities (37, 38), hence the concerns relating to top-down policy (14, 37).

To gain an understanding of the policy process, alongside the potential significance of a dedicated sport policy analysis framework, four mainstream meso-level frameworks commonly applied [see, for example (4, 26),] in sport policy analysis—the Institutional Analysis model, the Multiple Streams Framework, the Policy Network Theory model, and the Advocacy Coalition Framework—are discussed in light of the need to identify and address issues impacting sport. Said frameworks are considered as a result of the recent application in a sport setting (14, 26, 37). The frameworks are evaluated on the basis of their application in policy settings in general, followed by sport-specific examples to provide context and a point for comparison. That said, the evaluation of the frameworks is non-exhaustive—a complete analysis is beyond the scope of this paper—and the outline provided herein seeks to illuminate general aspects of each framework with a view to informing future research. With that context, this review is guided by the following research question: What influence do mainstream meso-level policy frameworks have on the sport policy process and the sustainability of grassroots sport?

## Method

Based on a qualitative approach, this paper seeks to provide a descriptive overview of policy frameworks commonly applied in the sport domain. To achieve this objective and to address the research question, the paper adopts a narrative review format review (39) and provides an outline of the four meso-level frameworks in a general context and their links to sport policy. Narrative reviews highlight connected events and describe topics from an essentially contextual position (40). Further, narrative reviews provide a succinct outline of literature and a platform to stimulate further research (41). Ultimately, with regard to the policy focus of this paper, the objective is to provide interpretation and critique intended to deepen understanding with a purposive selection of evidence (42).

### Data collection

The narrative review considered peer-reviewed journal articles and books/book chapters. The data was collected based on simple searches through Google Scholar and academic institution libraries, the latter of which allowed for specific search terms to be entered. No restrictions on time-frames were set, “gray” literature was not included, and articles with an overt focus on elite sport were excluded. The key search terms applied were *bottom-up policy*, *community sport, grassroots sport, meso-level, policy frameworks, sport, sport policy, and top-down policy*.

### Delimitations

In the first instance, this paper does not offer a comprehensive analysis. As a narrative review, the purpose is to provide a descriptive, not systematic, outline of the situation. Narrative reviews are not designed to be conclusive but instead provide a platform for further research (39). Unlike systematic reviews, for example, there are fewer guidelines governing narrative reviews (253). Researchers choose what should be the focus of the review and what will be included in order to offer useful research (255). Indeed, such literature reviews are almost always selective, non-exhaustive, and involve less demanding search methods (39).

## Literature review

The narrative review is divided into five sections consisting of Policy Background, the Institutional Analysis (IA) model, the Multiple Streams Framework (MSF), the Policy Network Theory (PNT) model, and the Advocacy Coalition Framework (ACF).

### Policy background

Historically, analyses of sport policy have addressed a range of matters including political issues such as foreign affairs, developments relating to governance, and concerns relating to equality, such as people with disabilities (43) and, more recently, issues relating to physical fitness and health, participation in sport, and general welfare (26). The scope of policy analysis includes analysing data on government policy intentions and the efficacy of government initiatives (4, 19, 44). Such analysis has consistently stressed the fact that government investment in sport is inconsistent (45–47), yet given government policy on health and participation, sound policy processes and evaluation might be considered essential to sport policy architects (6, 12, 48). Consequently, although government intervention in sport policy, including defining strategies and subsequent evaluation, has been the subject of academic enquiry, few analyses have addressed sport policy effectively (14, 49). In that context, a greater awareness of the stages of the policy-making process and the nature of policy subsystems—defined by the diversity and number of stakeholders and their policy focus (50–52)—can help policymakers leverage sport's inextricable role in society (12, 53, 54) and to address issues pertaining to macro-political agenda-setting (37).

From the perspective of understanding sport's relationship to society—its social role, especially at grassroots level (54–56)—it is useful to recognize the utility of mainstream analytical frameworks in the policy process, particularly to appreciate why the theoretical constructs of sport policy are based on the importance of meso-level assessment, as opposed to macro or micro-level approaches. An initial consideration is that sport policy serves aspects of more general public policy, particularly concerning community policy matters, where meso-level frameworks are commonly applied (57–59). Labelled as “policies that originate within, or are dependent upon the resources of, the state” (4), public policy significantly shapes sport policy (37, 60). Informed by macro-level theorising and micro-level evaluation, the direction for robust analysis is linked to policy analysis at the meso-level (61, 62). All meso-level frameworks incorporate macro-level assumptions, and when considering an analytical framework, any structure also needs to adequately cater for matters of stability and change (62, 63), no less so than in the sport policy process (64). Indeed, the scope for expounding the process of change, and a drive toward stability, is of particular importance in any analysis because there is evidence of regular and rapid change in sport policies and subsequent objectives globally (4), and in Australia (47, 65–67).

In identifying and illuminating numerous variables that make up the creation of policy, several analytical frameworks have an explicit focus on key stages of the process but are lacking when investigating the connection, and often interdependency, between key stakeholders (4). Further, there is consideration of contemporary policy-making and (a) assessing the nature of application of meso-level frameworks across various disciplines (4) and (b) that the frameworks have been subject to germane level of examination of their value as models for sound analysis of the policy process (62, 68). Sabatier (68) and Houlihan (4) provided a benchmark to gauge the merit of a meso-level approach to policy analysis, as per Table 1.

**Table 1:** Key criteria for meso-level analysis [soure: (4, 68)].

Sabatier	Houlihan
Explain policy stability and change based on values and interests, institutional arrangements, and socio-economic variations.	Have the capacity to explain both policy stability and change, which is of particular importance the sport domain.
Have a positive theory to explain much of the policy process.	Have the capacity to illuminate a range of aspects of the policy process.
Confirm their value and suitability as models and frameworks that credibly analyse the policy process.	Have applicability across policy areas beyond sport to cater for the distinctive features of sport policy.
The propositions of each framework must be constant, identify clear causal drivers, and apply to policy processes over a decade.	Facilitate a medium term (5–10 years) historical analysis of change because a shorter duration limits understanding.

Sabatier's (68) model supports generic policy analysis, whereas Houlihan's (4) version provides detail relating to sport; there is an emphasis on the conditions of stability and change in sports-related policy (64) due in part to “constant reviews of sports policy in many developed countries” (4). Such a scenario is in keeping with policy review processes in non-sporting disciplines in general which are very much subject to regular evaluation (69, 70). Indeed, policy processes and systems are complex (68, 71), challenged by a range of variables that disrupt the policy process. Such variables include (i) numerous and diverse stakeholders; (ii) timeframes for policy creation, implementation, and evaluation; (iii) involvement of government and the impact of strategies and objectives; (iv) conflict relating to issues and effect of policy change, and (v) coercive relationships directing the policy process (68). Due to the challenging nature of policy-making (14), such wide-ranging variables make it extremely difficult for the analysis to fully address every aspect of the process. In the following section, IA, the MSF, PNT, and the ACF, are outlined for understanding and addressing policy with a particular focus on policy implementation in sport and the impact of political, social, and cultural structures on the policy process (14).

### Institutional analysis

Applied extensively in the policy realm, IA represents a robust meso-level framework that examines institutional pressure on stakeholder conduct by way of a range of regulations and behavioral norms (72–74). Institutionalisation embodies a phenomenon through which stakeholders consent to a collective view leading to legitimisation, which occurs “whenever there is reciprocal typification of habitual actions by types of actors [stakeholders]” (75). From the perspective of research into the policy process, institutions form a central aspect of the arena within which policy-making takes place (63). IA provides estimable understanding of the role of institutional guidance—and coercion—and the resultant behavior of stakeholders (76); indeed, a key attribute of IA is that it facilitates the concurrent evaluation of stakeholder conduct and the establishment in which they operate (77, 78). The formation of an institutional collective relies upon the (a) resources brought to a situation, (b) value assigned to actions, (c) way knowledge and information are acquired and used, and (d) processes and justification for particular courses of action (76). In turn, the realisation of a collective accelerates the concept of an institution (79–81), particularly when considering the institutional nature of sport, both globally (82–87) and in Australia (66, 88–90).

In light of the multiple variables involved, a sound approach for the interpretation and role of institutions in the policy process is necessary (4). Institutions can be defined as (a) organisational entities such as government agencies (e.g., the Australian Sports Commission) and (b) cultural constructs with values and beliefs amongst stakeholders (4). Further, institutions occupy an official place from a legislative or procedural perspective, and are also seated more informally in relation to behavior or customs (76). Within that context, IA focuses on adapting the institutional development process, alongside the reciprocal stakeholder values and beliefs, to resolve policy issues (14). Individually, or as an element of collective, official settings, IA provides an avenue for connecting two or more individuals to align behavior and subsequent action (91, 92), and can help “elucidate the role institutions play in the determination of social and political outcomes” (93).

In terms of the application in a sport setting, IA has been utilized in macro-level analyses, reflected in sport policy literature (94), where the centrality of institutions to the policy-making process is noted (63), and where the significance of the function of institutions in sport policy processes is documented (4, 95–97). O'Brien (98) incorporated IA in a discussion analysing stakeholders working together across unequal governance levels in sport. The imbalance stemmed from the status of government involvement in public policy-making and the attempt to foster a culture of evidence-based policy considerations (98). A noted aspect of the importance of IA in sport policy analysis is based on the reliance many sport organisations have on government resources (14).

Some examples of the strong IA link between government sport policy objectives include national sporting organisations in the United Kingdom (UK), such as Sport England and UK Sport, being heavily influenced by the government and its focus on “modernisation” of sport (99), alongside Scandinavian examples where competitive sport poses an issue from an organisational perspective, and where structural and institutional relationships present challenges for change because they are based on strong “egalitarian” values (100). Other examples of the application of IA include the evaluation of sport development processes and the contribution from political circles, inclusive of local government, and not-for-profit groups (101, 102), along with more recent examples of the conceptualisation of institutional change from an international perspective, and the increasingly topical evaluation policies relating to concussion legislation (103, 104).

At grassroots level, there have been changes in the philosophical foundation of policy together with a strategic shift resulting in the prioritisation of one sport policy objective (e.g., elite sport) over another (e.g., grassroots sport) and the subsequent variation in levels of commitment from different institutions (23, 76). IA offers the potential to commence a process for identifying the key issues confronting grassroots sport (64, 105–108) and within that setting, there is merit in taking grassroots sport clubs as the point of departure in the policy process to build on the sociological perspective of IA (16). Finally, the outcomes emanating from IA are useful in “explaining policy related [sic] actions, interactions and outcomes” (109). Based on such observations, institutions represent independent and intervening variables in the policy-making process (4), a delineation that impacts policy implementation in relation to the differing priorities of implementers vs. government. Indeed, centrally designed policy influences localized implementation, potentially catering for sometimes contradictory but not mutually exclusive perspectives, and offers a potential solution for analysing the implementation process (16).

There is, however, a view that is problematic for IA which is that there is a lack of clarity about what an institution is and what institutionalism means (81). Ostrom's (76) reference to institutional pressures is validated by Thelen's (110) earlier argument that institutions meld the interests of stakeholders based on their relations of power with other stakeholders, which represents constraints and mediating factors in the political arena. Fischer (111) stated aspects of IA pointed to attempts to discreetly manipulate power within institutional structures, and Ostrom (76) recognized numerous organisations were governed by regulations which dictate patterns of interaction for organisations. Cairney (81) proposed that institutions are both the physical structures in which policy is made but also the rules that dictate behavior and how policy is made, with IA providing for a more robust emphasis on regulations, norms, and actions than on conventional arrangements.

The imprecision of IA is in part based on the variations in interpretation, whereas some uncertainties could be dismissed if IA was excluded as a “unified body of thought” (93). From a policy framework perspective, further problems arise because each new framework is perceived as a novel approach to the concept of institutionalism (93). In addition, ensuing deliberations create problems for the theoretical contribution that IA offers because its strength may lie in its multi-theoretical character (112). Thus, rather than strict distinction or disagreement between each version, IA may allow for the assessment of competing propositions drawn from different theories (81, 113). Houlihan (4) points to an historical context to IA, noting the autonomous nature of institutions, which contrasts markedly with resultant constraints enforced on society (114), viz., institutions can restrict options by influencing stakeholder perceptions and interpretations of problems and solutions (4, 115). Further, IA offers a mechanism for the control stakeholder behavior and expectations by addressing issues with generally appropriate methods (116).

Overall, the main disadvantage of IA is the explicit focus on various connected aspects of organisational structures, resulting in a reduced focus on institutional factors (94). In addition, IA generally only facilitates macro-level analysis (14), which is problematic if adopting a emphasis on all stages of policy creation and implementation, particularly the views of stakeholders implementing policy. Further, from a sport policy perspective, IA has been applied to analyse institutional conditions as opposed to technical settings (94) where, for example, the structure of sport organisations and the reliance on government resources results in a scenario where stakeholder behavior is influenced by more than institutional regulations and norms (14). Finally, the presumption that an institution might shape stakeholder opinions, behavior, and interests is potentially inaccurate for policy implementers involved with grassroots sport, where there is a lack of sophistication in terms of the process of implementation (117–119).

### Multiple streams framework

Established on a “garbage can” model of organisational choice (120, 121), Kingdon and Thurber's (122) MSF is theoretical paradigm applied in sport policy analysis (14). The concept of policy choice linked to the MSF leads to the garbage can analogy (123), where an array of policy issues, along with potential answers, are discarded (121). Although discussion and reason are fundamental to policy formation (124), the process comes with a degree of complexity where decision-making is characterized as a form of anarchy (125). The MSF emphasizes the chaotic character of organisations involved in policy creation, and political manipulation forms a significant part of the process (4, 51, 126). Indeed, there is a degree of ambiguity and accompanying attempts to regulate such uncertainty, in turn leading to governmental influence that “is more than just persuasion and identity construction” (125). Noting the manipulative and complex nature of the policy-making process (127), the MSF is made up of three distinct streams, namely, *problems*, *policies* and *politics* (51). Further, three additional conventions direct the MSF policy process: (i) individual processing is methodical and concurrent; (ii) there are substantial time limitations; and (iii) the three streams are separate (125).

Based on the key assumptions, the MSF provides an avenue to uncover the connections between individuals that manipulate policy processes, known as policy entrepreneurs, and individuals who are controlled as part of the policy process, the policy-makers (125). Policy entrepreneurs are “more than mere advocates of particular solutions; they are actors [stakeholders] that are power brokers and manipulators of problematic preferences and unclear technology” (125) who try to link the three MSF streams, which is contrary to their ordinarily level of independence (4, 51, 68, 99, 125). Further, policy entrepreneurs address disordered choices that have been created in an imprecise fashion, viz., a chaotic assortment of theories and concepts without any guidelines for implementation (121). Policymakers are those involved in making policies and policy decisions, often members of a government department, legislature, or head of an organisation responsible for making new rules or laws. Policy-makers can also be institutions, as opposed to individuals (128, 129). Often described as experts who consider their role to serve a cause greater than themselves, policy-makers face constant pressure on their cognition and emotion, and need to gather information quickly and effectively (130–133).

In relation to the three streams—*problems*, *policies* and *politics*—the MSF focuses on problems where public stakeholders want solutions, or where policy-makers deem action is required, in contrast to problems that are deliberately disregarded (4, 51). There is also the requirement for consensus to address issues, thus creating a catalyst for ensuring problems become part of a policy agenda (134). The policy stream incorporates a grouping of interrelated stakeholders pursuing a matter of public policy that is important to them for instrumental reasons (135). Alternatively, policy entrepreneurs embrace a particular issue and seek consent for solutions (4), and maintain the salience of a policy issue on the political agenda (95). The political stream occupies a position completely independent of other streams and includes national sentiment, political parties and lobby groups, and government (4, 51, 125). By observing general reactions and opinion, governments are better able to enact specific policy agendas or, conversely, dim the prospects of others (136).

In a sporting context, the MSF has been widely used in sport policy (14) and it is further positioned as a useful platform for sport policy analysis in nations worldwide and with diverse political conditions (4). In terms of policy agendas in sport, limited success on the international stage, declining participation levels, or rising obesity levels, serve as examples of common policy issues, especially based on mainstream initiatives. The scale of specific circumstances, potential for change, and success or failure of existing strategies are focal considerations within the MSF (125). Within that context, the success or otherwise of sport policy “can have a ripple effect through the political system by spilling over into other policy fields” (4), including situations where stakeholders focused on industry-specific initiatives are absorbed into more universal policy directives (60). The enunciation of inclusivity concepts within the sport policy system, however, reveals that sport policy is susceptible to manipulation by political, health and educational interests (137, 138). The matter of rising obesity levels serves as a topical example, demonstrating a direct correlation between declining levels of sport and physical activity and the resultant increase in expenditure on issues related to public health (14). Indeed, the MSF offers insight into the links between physical activity and public policy and helps facilitate a greater understanding of the problem and potential solution (139). Other examples of the MSF evaluation of policy change in physical education policy can be subject to the turnover of government ministers and general bureaucracy (140).

Changes in government and national sentiment can exert a considerable effect on policy agendas (125), pointing to the concept of how events of a political nature can focus attention on the policy process and create opportunities to legitimize sport and shape associated policy (14). By way of example, the accomplishments of British athletes competing at the 2008 Olympic Games in Beijing led to higher levels of government investment in the lead up to the 2012 London Olympics, alongside a general policy shift resulting in significant government support for the staging of an increasing number of international sports events in the UK. In Australia, following the success of the 2000 Sydney Olympics, the Federal government supported the 2016 Commonwealth Games in the Gold Coast and committed to underwrite fifty per cent of the costs for Brisbane to host the 2032 Olympic Games (141). Within that context, the three streams of the MSF link together at critical moments in time, wherein policy choices are made (125). Forming a critical stage of the MSF, the combination of the three streams leads to policy problems achieving recognition by government, and thus greater potential for a solution. For example, in sport there are potential favorable circumstances that relate to funding cycles linked to new polices to, for example, increase participation in physical activity through sport over a set period. Other examples include that of the Brazilian Olympic Committee (BOC) expanded investment possibilities by implementing policies that stimulated preparation for the Olympic and Paralympic Games in 2016 (142). From an MSF perspective, the BOC approach demonstrated the efficacy of the activities of policy entrepreneurs in the high-performance sport policy domain (142). In Australia, policy entrepreneurs have featured in grassroots sport, albeit subject to a complex array of factors internal and external to grassroots sport clubs, in various policy initiatives (13, 143).

In spite of the MSF's application by policy specialists, however, there are criticisms that relate to the assumptions of the framework and the limited perspectives of aspects of the policy process (125). These criticisms include a lack of unambiguous, falsifiable hypotheses within the MSF (62), and that the original “garbage can” model does not elicit robust findings which instead emanate from the MSF assumptions (144). Conversely, outcomes stemming from the MSF were arrived at due to the variations in each of the three streams, linked deliberately by policy entrepreneurs, and where data from different policy areas across various nations has been observed (51, 145, 146). The appropriateness of conceptualising independent streams, however, is debatable and the MSF streams can be deemed mutually dependent, with one prompting variations and modifications in the other(s) (123, 144).

Accordingly, the MSF's aptness for sport policy analysis is brought into question, as evidenced by its limited application (60, 127). Although the framework offers some direction in the policy analysis, that is largely only from a macro perspective, thus impacting its appeal to sport policy research. As a result, the MSF provides limited interpretation of matters relating to policy stability and change (99), particularly due to the claim that the MSF does fully assess policy beyond the initial opportunity (147), instead focussing mainly on formation and disregards other steps, notably matters of implementation (4, 14). Finally, timeframes for change are not an explicit consideration, there is overt attention on the notion that institutional foci influence political agendas (4), and there is an absence of empirical evidence (121). Ultimately, the capacity of the MSF to formulate and assess the complete policy process in sport is constrained; the “model focuses on policy issues, political intervention and solution development” (14).

### Policy network theory

PNT provides an avenue to assess numerous variables involved in policy change and the policy-making process as part of policy communities. As an integral component of PNT, a policy community is based on restricted, durable, and interdependent relationships, based on common values and beliefs amongst stakeholders, that form a homogeneous structure with joint accountability for policy implementation (14, 148). In terms of key stakeholders, governments frequently represent the dominant party in a policy community, at the expense of other non-government stakeholders (135, 148). Additional literature relating to PNT suggests it is suitable for investigating the impact on network stability in sport policy processes (149–151), and has the potential to provide a mediatory function between government and non-government stakeholders (152). Although a significant methodology for the analysis of public policy-making (153, 154), however, PNT lacks a single, accepted definition (63, 154, 155). Further, PNT has evolved as a result of undue government influence in the policy process (156), a situation that neglects the views of other stakeholders and the relationships between them (135).

The PNT approach builds on several theoretical frameworks that offer explanatory power of policy networks, both on the level of strategic interaction processes as well as on the level of institutional relations. Policy networks rely on “dependency relationships that emerge between both organisations and individuals who are in frequent contact with each other in particular policy areas” (157). Accordingly, PNT assumes that stakeholders that form a policy community are reliant upon each other to combine resources to achieve objectives (14). Ultimately, this situation benefits each stakeholder in reaching their goals based on mutual understanding (158). Within such policy communities, interaction between non-governmental stakeholders is largely egalitarian (159) and based on “stable patterns of social relations existing between interdependent actors [stakeholders], which take shape around policy problems and/or programmes” (61). There are four key elements that form the basis for PNT: (i) rational decisions, (ii) individual relations, (iii) formal analysis, and (iv) stable structures (160–162).

Understanding the nature of relationships in PNT is impacted by the way power is used, particular in relation to influencing government decisions (135). From the perspective of managing the balance of power, the interaction between stakeholders has led to numerous considerations for influencing the policy process, including the aforementioned policy communities, the concept of iron triangles—constraints based on budget, scope and schedule (163)—and focused issue networks (135). Thus, incorporating the view of power, PNT caters for the incorporating the nature of membership, stages of integration, sharing of resources, and levels of control, in the development of network types (148). Such viewpoints have methodological flaws, including what potentially amounts to overclassification, with designations for one set of circumstances being interchanged and applied to describe completely different phenomena (164). To address the inconsistency, a two-dimensional approach was developed based on dual factors: stakeholders and their interrelations (164). In terms of explaining these factors, there are two key variables: (i) composition element—the characteristics of stakeholders; and (ii) structural element—the forms of stakeholder relationships (164). Alongside the categorisations, the policy area of a network—“the territorial and functional specificities” (164)—incorporate political considerations that impact the ratification and structure of a policy network.

In sport, policy networks are often focused on altering the balance of power in order to influence the policy process by, in part, separating policy discussions from government agendas (14). Such manipulative objectives are often a result of relationship instability amongst unsuitable stakeholder numbers, resulting in the limited application of PNT in sport policy (14). An initial example relates to attempts in Australia, Canada, and the UK to analyse and elite sport policy programs based on global developments (165) where countries “interpret and adapt external policy pressures to their particular national circumstances and history” (166). Further, examination of the status and potential homogenisation of international sports policy results in policies being introduced internally, as part of a process of incorporating sport policy into national objectives (166). Other examples include the application of PNT to understand partnerships and policy development processes in the UK relating to physical education and sport programs, where various partnerships impacted policy outcomes (150). Also, PNT was applied to develop a new form of governance in UK sport to address aspects of interference in education and sport policy communities (167).

Although it is suggested that PNT can assume a mediatory function between government and key stakeholders (152), the model is criticized for its complex approach by combining multiple policies as part of the analysis. While there is the potential to apply top-down and bottom-up processes to provide a more thorough analysis, there is a risk that individual policies are not adequately assessed (158). In addition, the meso-level policy PNT approach creates a need for greater consideration of potentially complementary theoretical application (152), but introducing or merging other macro-level paradigms does not guarantee a sound analysis. Further, such investigation focuses on the associations between stakeholders as opposed to having a focus on the implementation and the consequences of policy; PNT does not have the capacity to address the steps that follow policy implementation (14). There are other issues that relate to PNT that are sometimes characterized by the diverse range of stakeholder interests (148) and on occasion viewed as volatile, unstable, and associated with policy consultation rather than the development of policy (148, 162). Although the categorisation of networks emanating from PNT is often helpful when evaluating various types of stakeholder nexuses at different levels, such methods usually represent radical stakeholder connections, and the practical application does not allow for a comprehensive approach (168).

### Advocacy coalition framework

Emerging in the 1980s, the ACF has featured in studies of public policy in many developed nations—both Western and Eastern (68, 169–171)—and increasingly so in developing countries (172, 173). The ACF is a contemporary meso-level framework in the policy realm, intended to expand policy analysis by incorporating a greater range of processes than other frameworks (4, 50, 52, 174–178). The framework provides a method to examine the behavior and attitudes of numerous policy stakeholders by clustering them into *advocacy coalitions*, formed by a range of stakeholders with common values, beliefs, and perceptions of policy issues, and who establish levels of coordination over time (179).

As an instrument of collective public action designed to address a public problem (180), the ACF is viewed as holistic, with a broad, multi-level focus on the policy process (50, 52) and has been applied in various domains and in numerous regions globally (176, 181, 182). The influence of the ACF on public policy development, implementation and analysis has been significant and has changed and shaped thinking about public policy (183, 184) based on the examination of policy change, policy learning, and the structure and stability of coalitions (147, 172, 184, 185). According to its founder, Sabatier (186) posits that the ACF has at least four basic premises: (i) policy change can take ten years or more; (ii) policy change occurs through subsystems involving the interaction of stakeholders seeking to influence government; (iii) the subsystems involve all levels of government; (iv) policies are conceptualized in conjunction with beliefs (186).

The format of the ACF is based on three key foundations, as follows: (i) a macro-level understanding that policy-making is based on the stability of behavior of key stakeholders in a policy subsystem is; (ii) a meso-level assumption that analysing stakeholder behavior is best achieved by clustering them into advocacy coalitions; and (iii) a micro-level approach to the beliefs of each stakeholder in a policy subsystem (50). The make-up of an advocacy coalition relies principally upon the alignment of beliefs which in turn generate the enthusiasm necessary to stir stakeholders into action—such stakeholders often engage in political activity to translate beliefs into action (184)—and it is the fundamental beliefs of implementing stakeholders involved in policy that lead to understanding the policy implementation process. The ACF encourages a fusion of top-down and bottom-up elements to better understand key aspects of the policy process (187–190). The top-down perspective is based on the influence of dominant parties in policy creation process and the fulfilment by those stakeholders responsible for implementing policy. The bottom-up approach argues that a sound process relies upon effective policy implementation; thus, the policy process should commence at the bottom and influence policy creation from that position (191).

Conventionally, a top-down approach to the policy process is based on a centralist model of policy creation and suggests that the likelihood of success at the implementation stage is based on the ultimate compliance of those implementing policy (192–194). In opposition to the top-down method, bottom-up theory focusses on the perceptions of grassroots stakeholders, or “street-level bureaucrats” (195, 196), the logic being that stakeholders responsible for implementation are also key to policy outcomes. Further, the bottom-up approach is based on the skills and abilities of stakeholders to arrive at a consensus, based on common values and beliefs, to constructively impact the environment and the associated implementation process. Supporters of bottom-up theory argue that it is a more realistic and progressive approach due to the focus on the perspectives and subsequent actions of street-level implementers (191). The decisions and routines of stakeholders responsible for policy implementation are determined by the burden of said implementation, a situation that is more challenging with a solely top-down policy process. Such conditions result in part from the paradox of the grassroots, street-level implementer: while the actions of policy implementers need to be orchestrated to realize policy aims based on the nature of policy creation as part of the political process, the effort of implementers also requires improvisation and responsiveness to individual circumstances (74).

In terms of examples in sport, the ACF has been operationalized by several researchers (175, 177, 197, 198) with numerous specific attempts to evaluate sport policy through the ACF (16, 199, 200). Significant research has been dedicated to the varying levels of commitment from the government to, for example, elite sport vs. grassroots sport (201). In their analysis of Canadian and UK examples, Green and Houlihan (177) found the ACF useful in understanding the process of change and policy making in elite sport. Further, the analysis of policy subsystems highlighted complex dealings between the respective national governments and sport organisations seeking to advance systems for elite sport, and it was noted that stakeholder beliefs were crucial in the process of change occurring over many years. The element of time, as per Sabatier (186), was noted by Houlihan and Green (99) where the changing status of sport policy in schools in the UK was analysed. In the European Union (EU), Parrish (202) assessed aspects of law relating to sport and identified two advocacy coalitions; the Single Market coalition, the dominant sport policy subsystem in the EU that, based on clear capitalist foundations, supported the commercialisation of sport, along with a socio-cultural coalition responsible for influencing sport policy generally (202). More recently, Fahlén and Skille (175) applied the ACF to assess policy processes impacting Sami sport, in Norway, and Yilmaz (198) examined the beliefs of coalition stakeholders in the EU and issues regarding aspects of integrity relating to agents in sport.

Despite the above examples, there are numerous challenges associated with the ACF in policy analysis. Firstly, the framework lacks sound consideration of the influence of institutions in the policy process (16), a potentially substantial oversight when considering institutional influence on stakeholders (203). Within that context, belief systems are considered more important than institutional affiliations (62), giving rise to a problematic view that belief systems do not guarantee a robust foundation for interaction within an advocacy coalition (14). Although a coalition may engage in activities in a synchronized fashion to achieve a common goal, they may not be not “jointly agreed upon” (204), such as monitoring other stakeholders' resources or strategies and shifting their behavior accordingly (205).

With issues linked to harmonising stakeholder beliefs within an advocacy coalition, it is difficult to identify a pathway for a coordinated approach to policy implementation at ground level. For example, beliefs stemming from grassroots sport issues would likely conflict with elite sport policy beliefs, thus impacting the potential for policy stability as part of a coalition structure. The ACF implies that stability relies upon “dominant coalitions and the persistence of deep core and policy core beliefs” (4). Such a platform, however, is critiqued for its hierarchical focus on the stability of belief systems in a coalition and the perception of consistency across all stakeholders. Schlager (206) argued that not all stakeholders in a coalition will have common beliefs; rather, it is potentially more realistic to assume that stakeholders may be able to coalesce, they can equally have divergent opinions. Further, the ACF's description of policy change relies upon a multifaceted interrelationship between cogent norms, external events, and policy learning, but fails to adequately account for the concept of power impacting coalition structure and relations (4).

Finally, while the ACF theoretically caters for a comprehensive analysis of policy-making, it does not properly outline multi-tier influence, viz., the impact of local, regional, and national involvement, particularly of government, on the policy-making process (4). Overall, there are issues with the ACF cultivating a comprehensive approach to the entire policy-making process and due to its meso-level application (50, 52, 207), policy input at some levels or from some stakeholders can be limited (4); thus, for example, clubs involved with grassroots sport—the stakeholders responsible for policy implementation—can be omitted from a potentially useful opportunity to influence the policy process (16).

### Review synthesis

Although there are numerous considerations, variables, and nuances when synthesizing the findings of this review, the scope of this paper is not to provide an exhaustive outline, rather to point to the overall influence of mainstream meso-level policy frameworks on the sport policy process. Accordingly, to provide contrast on key points, the review is broken down into a comparison between meso-level frameworks, as per Table 2.

**Table 2:** Key findings.

Framework	Positive Findings	Negative Findings
IA	• Places emphasis on institutional influence on policy processes • Identifies structural influence of policy actors in the policy process • Considers influence of policy actor behaviour and institutional structures	• Principally caters for macro-level policy analysis only • Relies on a combined theoretical approach to produce greater understanding • Assumes institutions predominantly influence policy actor behaviour
MSF	• Useful in identifying origins and stages of the policy process • Notable potential in a variety of different international policy settings • Points to clear links between problems and potential solutions	• The “garbage can” model fails to adopt a logical approach to a problem • Not considered holistic and does not adequately examine background to a problem • Does not fully recognize institutional influence
PNT	• Identifies role of interest groups and political influence • Considers influence of power in relationships between policy actors • Recognises interdependence of policy actors in the policy process	• Limited capacity for policy evaluation • Too great an emphasis on policy actor relationships instead of policy outcomes • Requires macro-level theories to complement approach to policy
ACF	• Useful for analyzing central policies such as national sport programs • Focuses on policy actor beliefs to influence policy change • Places emphasis on combination of top-down and bottom-up policy analysis	• Challenging to fully assess short-term policy objectives • Requires periods of circa ten years for sound policy analysis and evaluation • Considers belief systems to be more influential than intuitions

## Discussion

Sabatier and Weible (50) posit that the complicated nature of the policy process leads to a need for adequate tools to facilitate a comprehensive analysis. While the meso-level frameworks outlined herein may be considered, in part, robust means for analysis, their application is not without challenge (203). Further, many of the frameworks outlined fail to adequately consider the impact of policy, instead placing greater emphasis on policy creation, rather implementation and the outcomes thereof. In addition, each model brings into sharp relief the fundamental limitations relating to the evaluation of grassroots sport, where the implementation of policy occurs. With these points in mind, the main criticism herein concerns the boundaries relating to grassroots sport and long term, sustainable policy; the meso-level frameworks focus on the policy process leading up to implementation, as opposed to assessing and evaluating the outcomes to properly inform future policy creation. That is not to say that any single policy framework should or indeed can effectively cover all stages of the policy process, particularly with regard to nuances that arise in differing policy dynamics and in different disciplines [e.g., (57, 208)]. Within such context, however, the consideration of implementation theory as part of meso-level strategies because it is helpful to establish what happened but also the reasons why it happened (209) and interpret “what happens between policy expectations and policy results” (210).

Given the narrative herein based on issues associated with implementation, it is clear that criticism of historically dominant top-down policy approaches gives credence to bottom-up models that offer a real-world view of implementation and consideration of the reality from the perspective of the policy implementer (191). Based on macro and micro views of implementation (211), the bottom-up school outlines the nature of macro-level programs that are created, and subsequently, at the micro-implementation level, the policy morphs because local institutions adjust to the macro-level directives by implementing said programs within their capabilities (158, 211). A prominent characteristic of such situations, implementation-related issues result from the interpretation of policy by, and the limitations of, micro-level organisations. Dominant stakeholders responsible for creating policy, such as governments, have restricted power over, and access to, micro-level organisations, leading to the possibility for enhanced interpretation and understanding of change in relation to nationally formulated policy how it is implemented locally (211, 212). Such dynamic is an issue within the sport policy system and implementing a centrally created, one-size-fits-all policy (213) is fraught with challenge. Indeed, because contextual factors impacting grassroots sport are critical to policy processes and impact the nationally devised policy (158), if local policy implementers such as grassroots sport clubs are unable to manage policy edicts in a local context, the implementation process may be unsuccessful (158, 214, 215). Thus, at a micro-level, the beliefs of stakeholders involved with grassroots sport mean that policy subsystems will ultimately be comprised of stakeholders with a similar philosophical position (50, 52).

The investigation of the four meso-level analytical frameworks herein offers useful insight into a more pluralistic approach to sport policy. Indeed, there is good reason to consider the collective insights offered by IA, the MSF, PNT and the ACF, by operationalising multiple approaches to policy analysis and emphasising potential for theoretical pluralism in the policy process (184, 216). For example, Fahlén and Skille (175) posit that, for a more robust platform to conduct policy analysis in sport, blending the ACF with another meso-level framework, such as IA, would be advantageous. Galey and Youngs (217) suggest that, conceptually, a more thorough method for policy analysis might be realized by synthesising the ACF with other approaches to provide a more coherent theory of policy-making. With its acknowledgement of human agency, the ACF complements, for example, the emphasis on structures found in the IA approach (218). With a potential integration with IA, the ACF provides a strong theoretical framework for a comparative study in relation to the policy learning process. Incorporating IA into the approach will help to address analytically weak points of the ACF and such integration will provide valuable insights (219).

Green and Houlihan (177) suggest that the ACF offers only partial application for recreational sport policy analysis, adding that PNT—the “new paradigm of policy planning [that] focuses on governance processes which take place in policy networks or bargaining systems” (220)—is often favored due to its ability to embrace holistic analysis of the policy process. Adam and Kriesi (164), John (63) and Jenkins-Smith et al. (176) adopt a position whereby they link the ACF with PNT to promote a concept of analysis of policy processes that lead to a better understanding of applying more theoretical foci. With regard to the MSF, despite its weaknesses, Houlihan and Green (99) suggested that the MSF is potentially more beneficial compared to the ACF when examining school sport policy, and Jayawardhana and Piggin (14) also point to the reliability of the MSF for analysing sport policy. Finally, developing the concept of a blended approach to meso-level frameworks and accounting for old paradigms, a form of IA, along with an interpretation of the PNT, will merge with the ACF analysis; indeed, it is a “perspective that is deemed fruitful for the study of variations between local sport clubs and their relationships to central sport policy” (16). Applying a new paradigm of PNT at the level of individual policy subsystems within the ACF, alongside the integration of IA, there is the potential to better influence the decisions of government (221).

In sum, this evaluation of policy frameworks is worthy because it develops an understanding of the policy process—why it is important, how it works, and what the flaws are—and provides direction for change to policy (222) by enabling an interpretation of meso-level approaches that are more than a mere justification of policy (12, 37). Developing the comprehensive nature of the ACF (50, 52, 207) and with a sufficient level of stakeholder involvement—indeed, potentially myriad supporting stakeholders (68)—the ACF has a significant role to play in catering for grassroots influence on the policy-making process. Shared beliefs contribute to coalition stability (223, 224) and that forms a significant consideration for the sustainability of grassroots sport. Further, the ACF offers an avenue for the evaluation of policy objectives against more inclusive criteria that guides more effective policy creation and contributes toward policy learning (222). Such opinions are of specific significance to policymakers who need to manage increasing government interest in the governance of sport (4, 37, 225), especially when factoring in collaborative approaches to sport policy (27).

## Future direction

There is significant value in the sound analysis of sport policy, particularly the role of government, industry in general, the involvement of grassroots organisations, and the subsequent impact on longer-term strategic policy decisions (226). Within the context of formulating sport policy, any approach to planning needs to consider factors including policy structure, power relations, stakeholder beliefs, top-down and bottom-up methods, and the actions of implementers (227, 228). Given the lack of consensus on a universal method for sport policy analysis, however, and where developing one appears problematic (14), a potential opportunity for conducting reliable analyses might be to combine different theoretical perspectives (229). Indeed, creating a novel framework for evaluating sport policy that unites key advantages provided in existing frameworks has merit. There are, however, also suggestions that such an approach could lead to oversimplification of the policy process and that there remains a need for future research to direct greater attention to developing an holistic and ultimately sustainable framework for sport policy analysis (37). Thus, a key opportunity would be to consider further research—but of an applied nature in grassroots sport (225)—that focuses on policy implementation and gives greater credence to bottom-up policy influence including some of the key aforementioned factors of power relations, stakeholder beliefs, and the actions of implementers (227, 228).

As part of any further research, consideration will need to be given to the beliefs of key stakeholders involved with policy subsystems, the number of stakeholders, the diversity of their views, and their policy focus (50–52). Considering the Australian sport policy domain, for example, the different factors influencing the policy process is a point of deliberation due to the structural arrangements and multi-level governance practices. The diversity of stakeholders include all levels of government, government agencies, governing bodies of sport, academic institutions, health promotion organisations, and grassroots sport clubs, the implementers of policy (16). Examples of foci on strategic policy in Australia include the Future Directions policy which adopted an aspirational and inclusive approach to sport and recreation and priorities in terms of government decision-making processes (230). There are also the 2030 National Sport Plan and the Playwell policy which relate to strategies for sporting success and increasing physical activity in general (231, 232). In such a context, any consideration of meso-level frameworks needs to include a greater focus on understanding the implementation process (233, 234).

From an overall perspective of the policy process and the implementation issue, there is a *missing link* (31, 235–237) demonstrating that sound consideration of factors impacting implementation necessitates an understanding of inter-organisational dynamics, rather than an acceptance of solely top-down policy directives. In such scenarios, to understand the public setting where implementation ensues, and the viewpoint of those implementing policy, a bottom-up perspective provides an opportunity to consider government policy in the context of local conditions. Indeed, given the aforementioned notion that a robust approach to policy processes and evaluation might be valuable to sport policy planners (6, 12, 48), ensuring that the views of stakeholders in grassroots sport are factored into a conceptual framework that caters for more than a cursory influence on policy-making is crucial. Houlihan (4) advocates that for, policy for sport, the ACF “has a broader focus than many of its rivals and has the potential to illuminate aspects of the policy process beyond a preoccupation with agenda setting” (4), a position supported in more recent research involving the ACF (52, 175, 176, 178). With an evolving ACF theoretical model, organisations and governmental agencies might focus on their role in the policy-making subsystem and reduce the otherwise inherent and endemic organisational conflicts (238–240) that are reminiscent of a prior system.

With the above in mind, and despite the ACF's limitations, the relative use and consistent application of the ACF for policy analysis has significant potential (4, 165, 202, 224, 241). Further, the framework offers the possibility for innovation (242) and is well-tested in numerous policy domains and considered dependable in the sport policy realm (243), especially the notion of synthesising top-down and bottom-up policy processes (187–189). From the perspective of collaboration, a sophisticated and holistic approach is needed (37) in order to foster an environment conducive to more balanced and inclusive sport policy processes (244, 245). Without question, collaboration from a governance outlook lends itself to a more inclusive and potentially sustainable policy process (225, 246), particularly in sport policy (27).

In light of the absence of a definitive and dedicated meso-level for sport policy analysis and rather than reinventing the wheel or face the challenge of creating a more holistic policy framework, the ACF offers a robust and sophisticated construct for sport policy analysis, particularly from a more inclusive bottom-up perspective (175, 247). When contemplating a policy analysis framework to better understand processes impacting policy implementers involved with grassroots sport, the ACF lends itself to a potentially more universal approach (37, 62), and provides a valuable starting point as a tool for the analysis of sport policy (248, 249). Indeed, as Houlihan (4) suggests, the ACF offers a promising point of departure for a collaborative, inclusive, bottom-up, and ultimately sustainable approach to sport policy. Further, “before we discard a useful friend … we need to make sure … that we have a better, more robust framework on which to rely” (210).

## Conclusion

Through the lens of the ACF, incorporating the synthesis of top-down/bottom-up processes (187–189), the literature points to the potential for policy stakeholders to form a coalition with an holistic and more sustainable approach to sport policy (37, 62). With its emphasis on stakeholders, institutions and context, the ACF offers greater potential for policy analysis than other meso-level frameworks (4, 37). Based on a collaborative method and robust inclination toward bottom-up processes (250), there is the potential to identify if policy creators and implementers can better understand interactions between various levels of the policy process (particularly local), and accordingly, to appreciate if activity in one layer can act as a positive input into or influence upon other levels. Further, where grassroots, community involvement is important in local projects, the bottom-up process represents a superior consideration compared to the failings of top-down approaches (251). This is not to say that top-down processes will not be factored into a coalition structure—indeed, they must—and the analysis of the conditions preceding a coalition will dictate appropriate levels of emphasis on either a top-down or bottom-up approach (158, 252).

It is noted that for an ACF-based coalition to be effective, sport stakeholders may find it necessary to compromise and ensure member compliance (192–194). In that context, the ACF may not always incorporate what best suits all levels of sport policy and there is an element of *caveat emptor* when embracing the ACF (save that the same would be true for any coalition structure). Indeed, whereas the ACF remains helpful in the analysis and comprehension of shifts in values and beliefs and the link to policy change, alongside demonstrating that factors outside a policy subsystem require examination, there are numerous possibilities for superfluities to the framework for future analysis of sport policy. Flaws aside, however, the ACF is considered a practical and widely accepted structure for analysing the complete policy process (50, 52). The ACF meets key criteria for the purpose of analysing policy change (179) and is a valid approach within the arena of policy process research (181, 253). To reiterate Houlihan (4), the ACF is consequently a “valuable starting point for the development of analytical frameworks capable of illuminating the sport policy area” (4).
